# Image quality and evaluation ability of magnetic resonance imaging techniques for thyroid-associated ophthalmopathy: Dixon fat-suppression technique vs. spectral attenuated inversion recovery

**DOI:** 10.3389/fmed.2023.1154828

**Published:** 2023-07-12

**Authors:** Kai Huang, Xiaoxin Lin, Yaosheng Luo, Qiugen Hu, Baoliang Guo, Fusheng Ouyang, Yufeng Ouyang, Cheng Song, Haixiong Chen

**Affiliations:** ^1^Department of Radiology, Shunde Hospital, Southern Medical University, Foshan, China; ^2^Department of Endocrinology, Shunde Hospital, Southern Medical University, Foshan, China

**Keywords:** thyroid-associated ophthalmopathy, MRI, Dixon fat-suppression, spectral attenuated inversion recovery, image quality, lesion

## Abstract

**Purpose:**

We aimed to compare two magnetic resonance imaging (MRI) techniques, Dixon and spectral attenuated inversion recovery (SPAIR) fat-suppression, in terms of image quality and suitability for evaluating thyroid-associated ophthalmopathy (TAO) lesion characteristics.

**Methods:**

This cross-sectional, retrospective study involved 70 patients with TAO (140 eyes) who underwent orbital coronal MRI examinations, including Dixon-transverse relaxation (T2)-weighted imaging (T2WI) and SPAIR-T2WI, between 2020 and 2022. We compared the fat-suppression quality and artifacts, noise (N), signal-to-noise ratio (SNR), contrast-to-noise ratio (CNR), signal intensity ratio (SIR) of extraocular muscles (SIR-EOM) and lacrimal glands (SIR-LG), and TAO activity evaluation efficiency.

**Results:**

Dixon-T2WI showed a higher frequency of better subjective image quality and suitability for evaluating the characteristics of TAO lesions (65.7% vs. 14.3%) than SPAIR-T2WI. Fat-suppression quality and artifact scores were lower for Dixon-T2WI than for SPAIR-T2WI (*p <* 0.001). The N, SNR, and CNR values, EOM-SIR, and LG-SIR were higher for orbital coronal Dixon-T2WI than for SPAIR-T2WI (all *p* < 0.001). Clinical activity scores (CASs) showed positive correlations with SIR. The correlation between EOM-SIR and LG-SIR of orbital coronal Dixon-T2WI with CAS was higher than that of SPAIR-T2WI (0.590 vs. 0.493, all *p* < 0.001; 0.340 vs. 0.295, all *p* < 0.01). EOM-SIR and LG-SIR of Dixon-T2WI yielded a higher area under the curve than SPAIR-T2WI for evaluating TAO activity (0.865 vs. 0.760, *p* < 0.001; 0.695 vs. 0.617, *p* = 0.017).

**Conclusion:**

Dixon-T2WI yields higher image quality than SPAIR-T2WI. Furthermore, it has a stronger ability to evaluate TAO inflammation than SPAIR, with higher sensitivity and specificity in active TAO staging.

## 1. Introduction

Thyroid-associated ophthalmopathy (TAO) is an autoimmune disease associated with thyroid diseases; it accounts for approximately 20% of adult orbital diseases and considerably affects the quality of life of patients (1). The TAO course is divided into active and inactive stages. The active stage mainly involves an inflammatory reaction of the orbital tissue, which requires treatment with anti-inflammatory drugs, such as glucocorticoids. Interstitial fibrosis, collagen deposition, and steatosis are dominant in the inactive stage, and anti-inflammatory therapy is usually ineffective (2). Magnetic resonance imaging (MRI) possesses high-spatial resolution characteristics and can clearly display the anatomical structure of the orbit and the corresponding signal intensity (SI). Therefore, it is extensively used to evaluate the activity of TAO (3–5). Different signal intensities reflect different histological characteristics of orbital tissue (2). The higher the SI in the extraocular muscle (EOM), the more serious the edema caused by the inflammation. The comparison of the SIs of different orbital tissues helps evaluate the activity of TAO, which is very important for diagnosis, treatment planning, and prognosis.

A large amount of adipose tissue is present in the orbit. In transverse relaxation (T2)-weighted imaging (T2WI), intraorbital fat masks the inflammatory signal of the orbital tissue and results in poor visualization of the lesions. Fat-suppression (FS) sequences can obtain more image information and are associated with better lesion contrast than conventional sequences (6). The complex anatomical structure of the orbit and its surrounding sinuses and eyeball movement make artifacts very noticeable; consequently, the FS effect is not good, and the image quality is often not ideal (7). Therefore, it is necessary to choose an FS technique that can improve the visualization and clarity of orbital tissues and lesions during orbital MRI examination.

Spectral attenuated inversion recovery (SPAIR) is an FS technique based on the difference in the precession frequency of protons in water and fat. It combines the advantages of short tau inversion recovery (STIR) and frequency saturation, uses a selective 180° insulation pulse (insensitive to the B1 field to ensure the accuracy of the reversal angle), and is insensitive to the inhomogeneity of the B1 field (8). Dixon-based techniques, which rely on the water–lipid chemical shift, acquire signals when the signal phases of water and lipid are the same and opposite. The water and lipid phase images can be obtained by calculations. Water phase images are obtained by FS using Dixon technology (9, 10).

The Dixon and SPAIR sequences are excellent FS techniques commonly used in clinics. However, to date, there is a random use of these two techniques in orbital scans of TAO patients, and fat suppression sequences are of great value in evaluating TAO activity, but their comparative study has not been reported. Therefore, in this study, orbital magnetic resonance (MR) images of 80 patients with TAO, obtained using coronal, water-only Dixon-T2WI and SPAIR-T2WI sequences, were compared for image quality of the two techniques in the orbit and their value in evaluating the activity of TAO, to select an excellent fat suppression technique for MRI examination of TAO patients, and to provide a certain research basis for the next step to explore the imaging criteria for TAO activity evaluation and efficacy prediction.

## 2. Materials and methods

### 2.1. Research and design

The study complied with the Declaration of Helsinki, approved by the Ethics Committee of Shunde Hospital of Southern Medical University (KYLS20220123), with informed consent from all participants. The clinical and imaging data of patients with TAO who underwent orbital MRI examination at the Shunde Hospital of Southern Medical University from January 2020 to July 2022 were collected retrospectively. The age, gender, thyroid function status, History of glucocorticoid therapy, clinical activity score (CAS), diplopia, exophthalmos, and orbital MRI scans of the patients were collected. Clinical data were recorded in Table 1. The clinical and imaging data of 70 patients (140 eyes) were collected, including 41 men and 29 women (average age, 42.8 ± 13.6 years).

**Table 1:** Clinical characteristics of the TAO patients.

Clinical characteristics	
Age (year)	42.8 ± 13.6
gender(Male/Female)	41/29
Smoker, *n* (%)	26 (37.14)
Duration (m)	6 (3–12)
Thyroid function (Euthyroid/Hyperthyroidism/Hypothyroidism)	14/54/2
History of glucocorticoid therapy (No/Yes)	57/13
CAS (Inactive group/Active group)	55/85
Disease severity(mild /moderate–severe/very severe)	31/35/4
diplopia (No/Yes)	59/11
exophthalmos (No/Yes)	7/63
Eyesight	0.71 ± 0.32
Intraocular pressure (mmHg)	19.43 ± 4.14

CAS, clinical activity score; CAS took each orbit as the basic study object.

### 2.2. Research object

**Figure 1:**
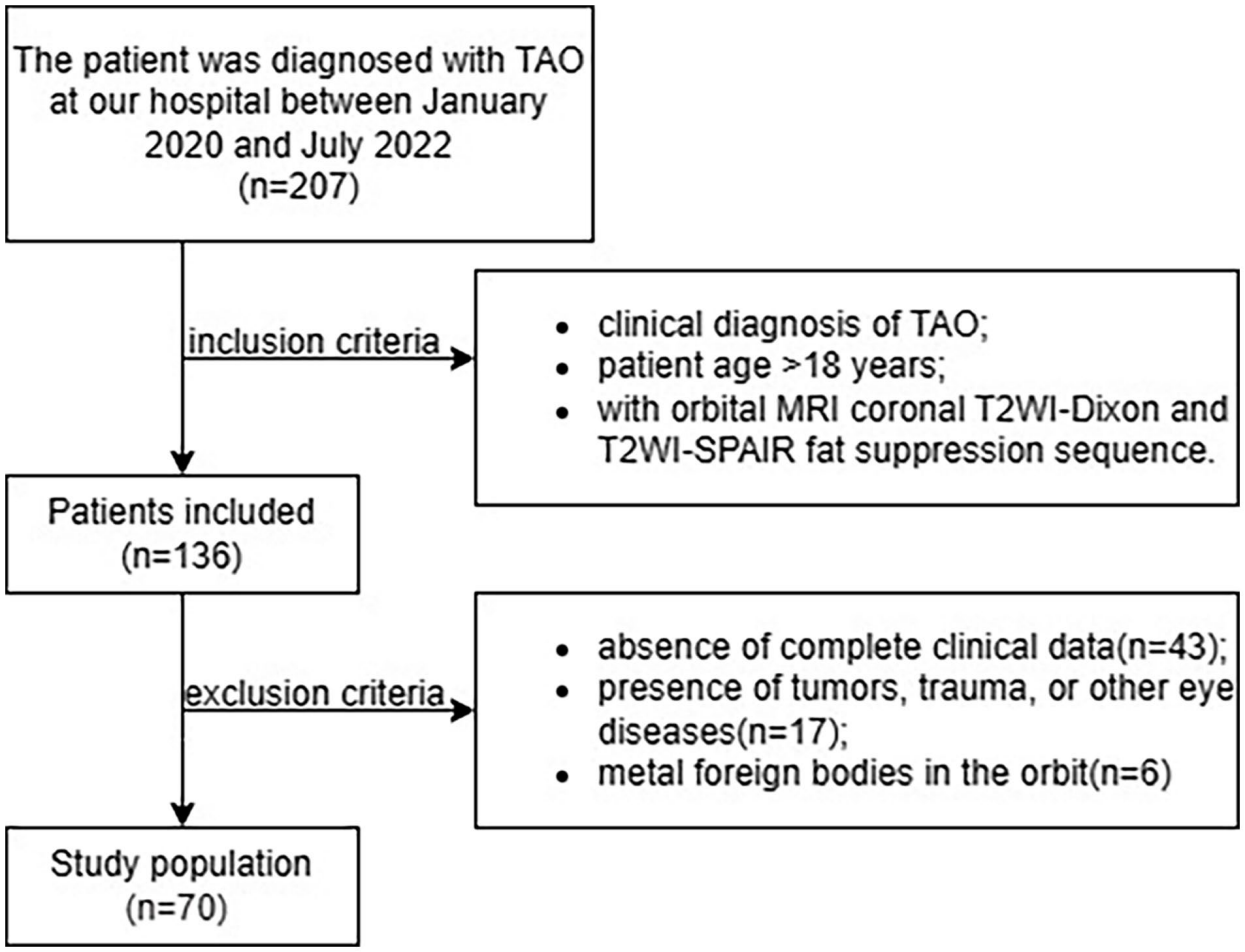
Flowchart of patient selection process. SPAIR, spectral adiabatic inversion recovery; TAO, thyroid-associated ophthalmopathy.

The inclusion criteria were as follows: (1) clinical diagnosis of TAO according to the EUGOGO guidelines and Bartley criteria (2, 11), (2) patient age > 18 years, and (3) availability of MRI data obtained with both Dixon and SPAIR FS sequences in the orbital coronal plane. The exclusion criteria were as follows: (1) absence of complete clinical data, (2) presence of tumors, trauma, or other eye diseases, and (3) metal foreign bodies in the orbit (Figure 1).

The CAS was obtained as follows (12): one point each was assigned for the presence of (1) spontaneous retrobulbar pain, (2) eyeball pain after activity, (3) eyelid redness, (4) eyelid edema, (5) bulbar conjunctival edema, (6) conjunctival redness, and (7) lacrimal caruncle swelling—a total of seven points. The scores were classified as follows: CAS ≥ 3, active stage and CAS < 3, inactive stage.

**Figure 2:**
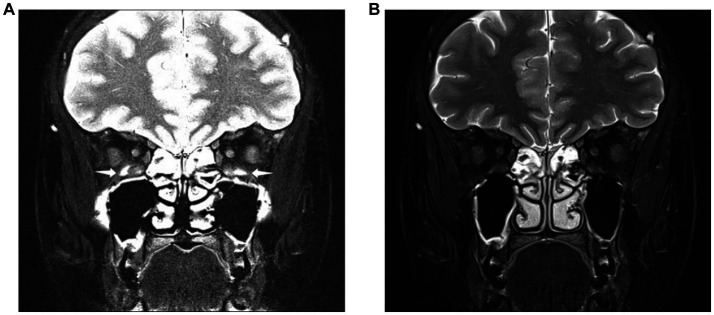
Images obtained from a 28-year-old woman diagnosed with thyroid eye disease. Coronal SPAIR-transverse relaxation (T2)-weighted imaging (T2WI) **(A)** and water-only Dixon-T2WI **(B)**. The latter image shows a more uniform fat suppression on the orbit and periorbital tissue than the former. Coronal SPAIR-T2WI **(A)** displaying the major susceptibility artifacts (white arrowheads) involving both inferior rectus muscles, hindering accurate analysis of muscular signal. Coronal water-only Dixon-T2WI **(B)** displaying the fewer susceptibility artifacts and strong fat-suppression ability.

**Figure 3:**
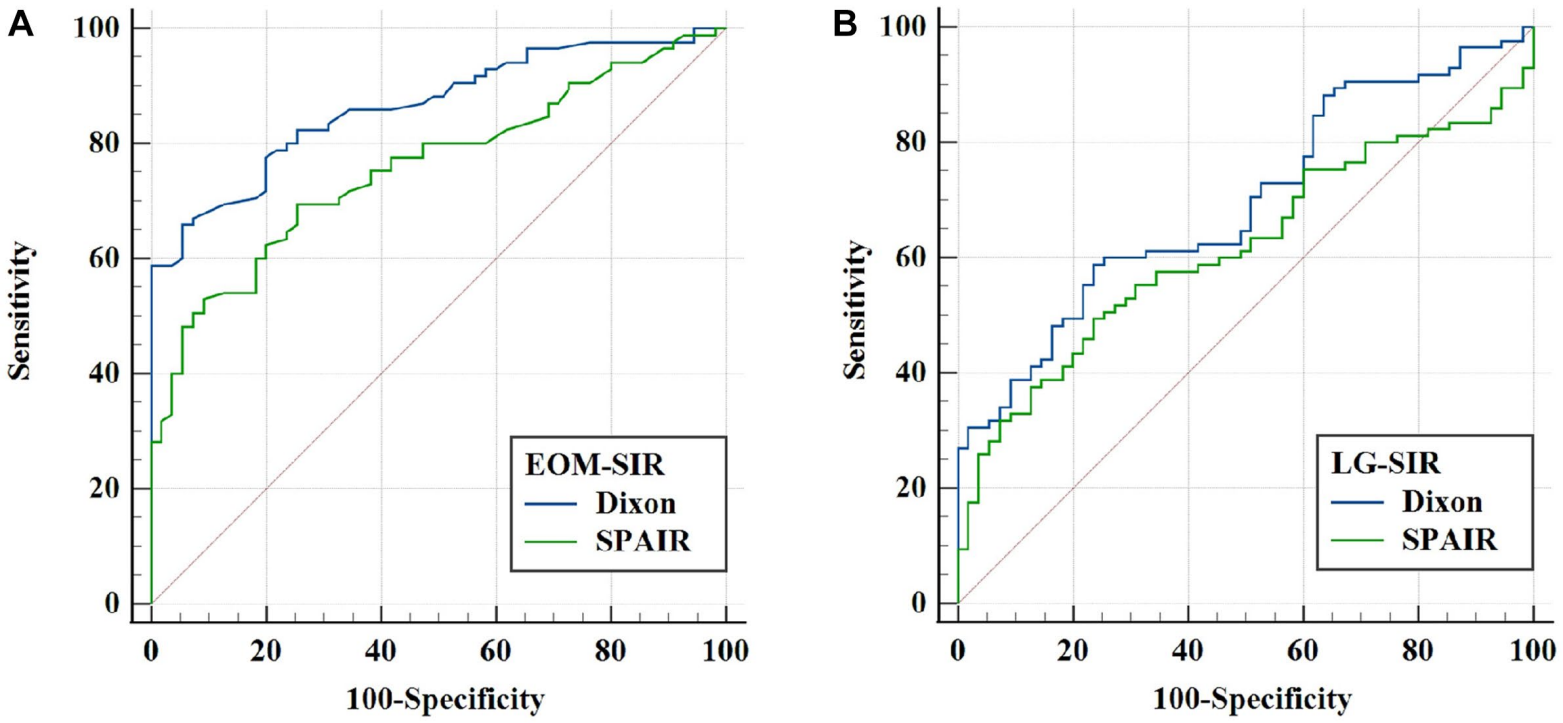
ROC analysis of extraocular muscle (EOM) and lacrimal gland (LG) SIRs for evaluating thyroid-related eye disease (TAO) activity. **(A)** Comparison of the ROC curves of EOM-SIR of orbital coronal Dixon-T2WI and SPAIR-T2WI in evaluating TAO activity. **(B)** ROC curve comparison of LG-SIR of orbital coronal Dixon-T2WI and SPAIR-T2WI in evaluating TAO activity.

### 2.3. MR examination

A Siemens Magnetom Skyra 3.0 T (Siemens Healthcare, Erlangen, Germany) MR scanner and a 20-channel phased array head coil were used to examine patients in the supine position. Patients were instructed to close their eyes and fix their eyeballs directly in front. The scanning range included the orbit and optic nerve. The scanning sequence included a conventional orbital plain scan sequence and coronal Dixon and SPAIR fast spin-echo (turbo spin-echo, TSE) T2WI. The specific scanning parameters are listed in Table 2.

**Table 2:** Imaging parameters of coronal Dixon-T2WI and SPAIR-T2WI.

	TSE Dixon-T2WI	TSE SPAIR-T2WI
Scan mode	2D	2D
Repetition time (ms)	4,000	4,200
Echo time (ms)	91	86
Echo train length	21	18
GRAPPA factor	2	2
Flip/refocusing angle	150°	150°
Number of excitations	2	3
Slice thickness (mm)	3	3
Gap (mm)	0.6	0.6
Pixel size (mm)	0.47	0.47
Field-of-view (mm)	150 × 150	150 × 150
Bandwidth (Hz)	381	260
Acquisition matrix	320 × 320	320 × 320
Acquisition duration	3 min and 32 s	2 min and 14 s

SPAIR, spectral attenuated inversion recovery; T2WI, transverse relaxation weighted imaging; TSE, turbo spin-echo; 2D, two-dimensional; GRAPPA, GeneRalized Actocalibrating Partially Parallel Acquisition.

### 2.4. MR image analysis

The coronal water-only Dixon-T2WI and SPAIR-T2WI data of each eye were analyzed. All parameters were measured using the Picture Archiving and Communication Systems of the Shunde Hospital of Southern Medical University (Yi Lianzhong Ruitu, Version 3.7, Guangzhou, Guangdong, China). Two radiologists analyzed the images independently by using the blind method and resolved disagreements through consultations. In our study, the subjective evaluation of image quality such as FS quality and artifacts of Dixon-T2WI and SPAIR-T2WI is conducted concurrently by radiologists (Figure 2).

### 2.5. Subjective evaluation

#### 2.5.1. Subjective evaluation of readers

The radiologists concurrently and subjectively evaluated the Dixon-T2WI and SPAIR-T2WI to determine the image quality and suitability for evaluating the characteristics of lesions.

#### 2.5.2. FS quality

The FS quality of images was classified based on a 0–4 grade scale (from good to bad): grade 0, appearance of complete FS in the scanning range, low-intensity signal from the fat-containing tissue on the fat-suppressed image, and distinct tissue structure; grade 1, slightly incomplete inhibition of subcutaneous fat and distinct tissue structure; grade 2, appearance of incomplete fat inhibition or dislocation separation of water and fat in non-important parts and retention of clear tissue structure; grade 3, appearance of incomplete fat suppression or dislocation separation of water and fat in important parts and unclear tissue structure to the extent that it affected the diagnosis; grade 4, poor fat inhibition of images in the scanning range, suppression of non-adipose tissue signals or occurrence of large-area fat inhibition or dislocation separation of water and fat, and unclear structure of each tissue to the extent that it affected the diagnosis.

#### 2.5.3. Artifacts

Image artifacts were classified based on 0–4 levels from good to bad: level 0, no artifacts; level 1, small artifacts at the edge of the image; level 2, artifacts that are obvious but do not interfere with the region of interest (ROI); level 3, prominent artifacts in the ROI; level 4, artifacts that are too severe to evaluate in the ROI.

### 2.6. Objective evaluation

Image noise (N), signal-to-noise ratio (SNR), and contrast-to-noise ratio (CNR) were calculated according to the methods recommended by the National Electrical Manufacturers Association (13).

#### 2.6.1. Measurement methods

(1) The section with the largest cross-sectional area of the EOM was selected on the coronal FS image, and ROIs were placed on the four corners of the image background, medial rectus, inferior rectus, lateral rectus, superior rectus, temporal muscle, and white matter, avoiding the areas of the cavity, blood vessels, and artifacts as far as possible, and an ROI was placed on the lacrimal gland (LG) in the section with the largest cross-sectional area. The average SI and standard deviation (SD) of the ROI were recorded; (2) N= $\bar{SD}$ /0.655, $\bar{SD}$ was the average of the ROI-SD values obtained from the four corners of the image background, and 0.655 was the noise correction factor (14, 15); SNR = SI/N and (3) CNR is the ratio of the SI difference to the noise of two different anatomical tissues.

#### 2.6.2. SI ratio (SIR)

(1) The ratio of SI of the EOM to the normal white matter of the ipsilateral homolayer (EOM-SIR) reflects the degree of inflammation of the EOM and (2) The ratio of SI of the LG to the normal white matter of the ipsilateral homolayer (LG-SIR) reflects the degree of inflammation of the LG.

### 2.7. Statistical analysis

All experimental data were statistically analyzed using the software *SPSS* (version 25.0, IBM Corporation, New York, United States). The subjective evaluation of readers is based on metric data expressed in terms of frequency and percentage. Data on the quality of FS and image artifacts were graded data, expressed as frequency and percentage, and compared between groups using the Wilcoxon signed-rank test. SNR, CNR, and SIR were used as metric data, and their normality was assessed using the Kolmogorov–Smirnov method. Normally distributed data were subjected to paired t-tests for comparison between groups and are presented as mean ± SD (X ± *S*). Non-normally distributed data were subjected to the Wilcoxon symbolic rank test for comparison between groups and are presented as median (lower quartile, upper quartile). Spearman correlation was used to analyze the correlation between EOM-SIR, LG-SIR of orbital coronal Dixon-T2WI and SPAIR-T2WI, and CAS. Taking CAS ≥ 3 and CAS < 3 as dichotomous variables, the receiver operating characteristic (ROC) curve method was used to analyze the critical value and prediction efficiencies of EOM-SIR and LG-SIR of orbital coronal Dixon-T2WI and SPAIR-T2WI to evaluate the active stage of TAO, AUC comparison by Delong’s test. The significance level was set to *α* = 0.05 (bilateral). A difference was considered statistically significant at *p* < 0.05.

## 3. Results

### 3.1. Readers’ subjective evaluation

The evaluators concluded that in terms of image quality and feasibility of evaluation, Dixon-T2WI of 92/140 (65.7%) eyes were better than SPAIR -T2WI, and SPAIR-T2WI of 20/140 (14.3%) eyes were better than Dixon-T2WI; subjective evaluation parameters of 28/140 (20%) only ocular Dixon-T2WI and SPAIR-T2WI were at similar levels.

### 3.2. FS quality and Artifacts

The FS quality score and artifact score of orbital coronal Dixon-T2WI were significantly lower than those of orbital coronal SPAIR-T2WI (*p* < 0.001) (Table 3).

**Table 3:** Comparison of fat-suppression quality and artifact scores (*n*%) between orbital coronal Dixon-T2WI and SPAIR-T2WI [*n* (%)].

Criteria	0	1	2	3	4	Wilcoxon sign-rank test	
						Z	*p*-value
Fat-suppression quality							
Dixon	15 (10.7)	70 (50)	25 (17.9)	26 (18.6)	4 (2.9)	6.632	<0.001
SPAIR	1 (0.7)	23 (16.4)	79 (56.4)	37 (26.4)	0 (0)		
Artifact							
Dixon	4 (2.9)	46 (32.9)	52 (37.1)	28 (20)	10 (7.1)	7.121	<0.001
SPAIR	0 (0)	9 (6.4)	41 (29.3)	80 (57.1)	10 (7.1)		

Data are presented as numbers (percentage) for the indicated numbers of patients in each group.

### 3.3. N, SNR, and CNR

N of orbital coronal Dixon-T2WI was significantly lower than that in orbital coronal SPAIR-T2WI (*p* < 0.001). The SNR and CNR values of the medial rectus, inferior rectus, lateral rectus, superior rectus, temporal muscle, white matter, and LG in orbital coronal Dixon-T2W were significantly higher than those in orbital coronal SPAIR-T2WI (*p* < 0.001) (Table 4).

**Table 4:** Noise, SNR, CNR, and SIR of the extraocular muscle and lacrimal gland for orbital coronal Dixon-T2WI and SPAIR-T2WI.

Criteria	Dixon	SPAIR	*p*-value
N	7.22 ± 2.75	11.65 ± 2.73	<0.001*
SNR			
MR	59.77 (44.45, 92.53)	32.48 (25.79, 42.73)	<0.001
IR	56.40 (37.41, 83.50)	32.33 (24.66, 42.73)	<0.001
LR	45.25 (32.21, 67.44)	23.61 (17.29, 31.20)	<0.001
SR	53.32 (41.57, 84.20)	29.44 (21.98, 41.62)	<0.001
TM	23.28 (17.68, 39.50)	11.99 (9.80, 16.06)	<0.001
WM	46.54 (34.54, 74.35)	28.22 (22.40, 33.55)	<0.001
LG	58.98 (39.83, 85.67)	30.76 (22.19, 38.14)	<0.001
CNR			
MR	37.07 (25.84, 53.95)	19.54 (13.87, 30.62)	<0.001
IR	31.25 (18.11, 43.93)	19.00 (13.61, 28.58)	<0.001
LR	20.18 (11.95, 31.48)	10.95 (7.21, 17.46)	< 0.001
SR	28.30 (18.00, 44.61)	16.48 (11.00, 27.54)	<0.001
WM	21.28 (15.46, 34.69)	14.89 (11.87, 19.07)	<0.001
LG	32.17 (21.46, 49.21)	16.76 (12.36, 24.48)	<0.001
SIR			
EOM	1.32 (1.10, 1.79)	1.27 (1.06, 1.68)	<0.001
LG	1.24 ± 0.33	1.12 ± 0.29	<0.001*

Data were presented as mean ± standard deviation (SD) or median (upper and lower quartiles) for the indicated number of patients in each group. *p*-values for comparisons between groups were based on paired t- or Wilcoxon signed-rank tests. *paired t-tests.

SNR, signal-to-noise ratio; CNR, contrast-to-noise ratio; EOM, extraocular muscles; N, noise; MR, medial rectus; IR, inferior rectus; LR, lateral rectus; SR, superior rectus; TM, temporalis muscle; WM, white matter; LG, lacrimal gland, SIR, signal intensity ratio.

### 3.4. SIR

The EOM-SIR and LG-SIR of orbital coronal Dixon-T2WI were significantly higher than those of SPAIR-T2WI (*p* < 0.001) (Table 4).

### 3.5. EOM-SIR and LG-SIR with CAS

Clinical activity scores (CASs) showed positive correlations with SIR. The correlation between EOM-SIR of orbital coronal Dixon-T2WI with CAS was higher than that of SPAIR-T2WI (0.590 vs. 0.493, all *p* < 0.001). The correlation between LG-SIR of orbital coronal Dixon-T2WI with CAS was higher than that of SPAIR-T2WI (0.340 vs. 0.295, all *p* < 0.01) (Table 5).

**Table 5:** Correlations of the signal intensity ratio of extraocular muscle and lacrimal gland with clinical activity score.

Criteria		*r*	*p*-value
EOM-SIR	Dixon	0.590	<0.001
	SPAIR	0.493	<0.001
LG-SIR	Dixon	0.340	<0.001
	SPAIR	0.295	<0.001

Spearman correlation was used to analyze the correlation between EOM-SIR, LG-SIR, and CAS.

EOM-SIR, ratio of signal intensity of extraocular muscle to normal white matter of ipsilateral homolayer; LG-SIR, ratio of signal intensity of the lacrimal gland to the normal white matter of the ipsilateral homolayer.

We also performed ROC curve analysis. Comparison of the corresponding parameters between the two FS techniques revealed the following findings: the area under the curve (AUC) of EOM-SIR of orbital coronal Dixon-T2WI were higher than those of SPAIR-T2WI (0.865 vs. 0.760, *p* < 0.001); the AUC of LG-SIR of orbital coronal Dixon-T2WI were higher than those of SPAIR-T2WI (0.695 vs. 0.617, *p* = 0.017) (Table 6; Figure 3).

**Table 6:** Predictive performance of SIRs of extraocular muscles and lacrimal glands in the differentiation between active and inactive thyroid-associated ophthalmopathy (TAO).

Criteria		AUC (95% CI)	Specificity %	Sensitivity %	*p*-value
EOM-SIR	Dixon	0.865 (0.807–0.923)	94.5	65.9	<0.001
	SPAIR	0.760 (0.682–0.838)	91.9	54.1	
LG-SIR	Dixon	0.695 (0.609–0.781)	76.4	58.8	0.017
	SPAIR	0.617 (0.525–0.709)	76.4	49.4	

CAS was treated as a dichotomous variable. AUC comparison by Delong’s test.

AUC, area under the curve; CI, confidence interval.

## 4. Discussion

In this study, Dixon and SPAIR techniques were used to evaluate the activity of TAO for the first time. The subjective and objective evaluation methods were used to compare and analyze the image quality of Dixon and SPAIR in the orbit, and to compare the effects of SIR of the extraocular muscles and lacrimal glands on the evaluation of the degree of inflammatory edema in the orbital tissue and the value in evaluating TAO activity. To explore the selection of an excellent fat suppression technique for MRI examination of TAO patients, and to provide a research basis for the next step to explore the imaging criteria for TAO activity assessment and efficacy prediction. This study shows that Dixon technology can not only improve image quality, but also improve the display of orbital tissue lesions, improve the detection rate of TAO extraocular muscle and lacrimal gland inflammatory edema, and has higher sensitivity and specificity in semi-quantitative evaluation of TAO activity stage.

**Table 7:** The signal intensity ratio of extraocular muscles and lacrimal glands in patients with inactive TAO was greater than the cut-off value.

Criteria		Number
EOM-SIR	Dixon	3 (5.6%)
	SPAIR	5 (9.3%)
LG-SIR	Dixon	14 (25.9%)
	SPAIR	14 (25.9%)

Data are number (percentage) for the indicated number of patients in each group.

The air spaces of the periorbital bone and paranasal sinuses often generate a magnetic susceptibility effect that leads to uneven FS and seriously affects the evaluation of orbital tissue signals, thus affecting the diagnosis of TAO. The results showed that the quality of fat inhibition by Dixon-T2WI was better than that by SPAIR-T2WI. Lee et al. (16) showed that the lumbar Dixon sequence was superior to the SPAIR sequence with regard to the quality of FS, which is consistent with the results of this study. This is because SPAIR is insensitive to B1 field inhomogeneity and has high FS selectivity, despite its frequency saturation and STIR disadvantages (17). However, SPAIR is sensitive to the inhomogeneity of the B0 field, which leads to uneven FS in anatomically complex parts of the orbit (18). The Dixon sequence is not sensitive to the heterogeneity of the B0 and B1 fields; therefore, the effect of FS is better. Wendl et al. (19) also confirmed in a head and neck study that the Dixon-FS technique is superior to the spectral technique because of the improved homogeneity of the FS.

The occurrence of magnetic susceptibility artifacts is closely related to heterogeneous FS (20). Because the complex anatomical structure of the orbit makes the local magnetic field uneven, FS is often uneven, and it is easy to produce prominent magnetic susceptibility artifacts. EOMs are distributed around the orbit and are easily disturbed by the inferior and medial recti near the maxillary and ethmoid sinuses, which leads to the development of artifacts. The most commonly involved EOMs in TAO are the inferior and medial recti (2). Therefore, it is very important to minimize artifacts during the evaluation of TAO. In our study, the artifact score of Dixon-T2WI was less than that of SPAIR-T2WI (*p* < 0.001). Lee et al. also reported that the Dixon technique shows fewer artifacts in the lumbar region than SPAIR, which is consistent with our findings (16).

In a study on skeletal muscle tumors, Huijgen et al. (21) found that the number of motion artifacts was slightly higher with the Dixon technique than with SPAIR. In our study, we also found that the Dixon-T2WI technique yielded a significantly higher number of motion artifacts in some patients than SPAIR-T2WI. However, other artifacts, such as magnetic susceptibility artifacts and chemical shift artifacts, were fewer. Therefore, the Dixon-T2WI technique yielded fewer artifacts than SPAIR-T2WI. Indeed, we can also reduce motion artifacts by informing patients to keep their eyes as still as possible and reducing the scanning time. The Dixon technique is associated with four types of contrast images (same phase, anti-phase, water phase, and fat phase) simultaneously, which is equivalent to the combination of FS images and T2WI scanning, which can save scanning time and improve scanning efficiency.

SNR is a quantitative basic index used to evaluate image quality (22, 23). Because the Dixon technique does not cause signal loss, as do other FS technologies, theoretically, it is associated with high-SNR outcomes (24). No previous studies have compared the SNR differences between the Dixon technique and other FS techniques with respect to the orbit. Kirchgesner et al. (25) showed that CHESS yields higher SNR than the Dixon-T2WI sequence. However, Zaike et al. (26) showed that the Dixon sequence has a higher SNR than CHESS. In this study, Dixon-T2WI has higher SNR and CNR than SPAIR-T2WI (*p* < 0.001), and SPAIR-T2WI has the advantage of high SNR compared with the frequency-saturation method. Lee et al. (16) also proved that Dixon-T2WI has a higher contrast ratio and CNR than SPAIR-T2WI.

We speculate that this may be due to the limited sample size of the study by Kirchgesner et al. and the continuous development and improvement of the Dixon technique; alternately, the SNR of the frequency-saturation FS technique is higher than that of SPAIR.

The SI on fat-pressing T2WI is closely related to the tissue water content, which is typically regarded as an index used to evaluate the intensity of tissue edema and inflammation. SIR can semiquantitatively evaluate the degree of orbital inflammation in patients with TAO and assess the activity of TAO (27). In this study, the Dixon-EOM-SIR and Dixon-LG-SIR were higher than those of SPAIR. Chen and Gaddikeri et al. found that the EOM-SIR of Dixon-T2WI was higher than that of other FS techniques (such as STIR, SPIR, and conventional fat saturation) (28, 29). This suggests that the Dixon technique provides high FS and highlights the contribution of tissue inflammation to SI. The high SI of edema caused by EOM and LG inflammation is more obvious in Dixon-T2WI. Therefore, the Dixon technique is superior to the SPAIR technique in the orbital inflammation evaluation. Ollitrault et al. (30) also found that the Dixon technique shows high sensitivity and specificity for evaluating orbital tissue inflammation.

Several studies showed that the SIR of EOM and LG is positively correlated with CAS, as consistently verified by the results of this study (7, 31). However, our study also showed that the correlation between EOM-SIR and LG-SIR and CAS in orbital coronal Dixon-T2WI was higher than that in SPAIR-T2WI, and the correlation between EOM-SIR and CAS was higher than that in LG-SIR. SIRs of EOM and LG can be used as quantitative indicators of evaluation activities, and EOM is the most sensitive. The higher the SIRs of EOM and LG, the more severe the inflammation of EOM and LG, the higher the TAO activity, and the higher the CAS score. Further analysis with the use of the ROC curve method showed that EOM-SIR and LG-SIR of Dixon-T2WI exhibited higher AUC, sensitivity, and specificity values than SPAIR-T2WI in assessing TAO activity. EOM-SIR was superior to LG-SIR in assessing TAO activity.

However, our study showed that the SIR of some patients was very high; however, the CAS was less than 3, which indicates that TAO was of inactive stage (Table 7). We speculate that the reason behind these findings may be that the clinical symptoms of inflammation of the deep orbital structure are mild, and inflammation of the retrobulbar tissue cannot be observed clinically. The reason for this more prominent phenomenon of lacrimal gland may be that the correlation between lacrimal gland inflammation and CAS is lower than that of extraocular muscles. Therefore, it is difficult to evaluate the inflammation of the retrobulbar tissue of TAO using the CAS score alone. MRI can identify inflammation of retrobulbar tissue more sensitively than clinical evaluation. In this study, the ROC curve method used CAS ≥ 3 as an index to evaluate the active stage of TAO; however, CAS could not accurately reflect the activity of TAO. It is possible that SIR combined with CAS can more effectively evaluate the degree of orbital inflammation and TAO activity. However, this hypothesis needs to be confirmed in future research.

Our study had several limitations. First, this was a single-center retrospective study and may involve a selection bias. Second, our sample size was limited because we selected patients who underwent both coronal Dixon-T2WI and SPAIR-T2WI. Third, no contrast-enhanced scans were obtained in this study. Fourth, we used only one 3.0 T MRI for scanning, and the imaging results of Dixon-T2WI and SPAIR-T2WI may differ among different MRI parameters and field intensities; however, we adjusted the image parameters as much as possible to obtain better image quality. Fifth, our study lacked pathologic evaluation results. Finally, this is a purely subjective study in terms of image quality assessment of fat suppression and artifacts.

In conclusion, our research showed that the Dixon technique yields higher image quality, better FS, and fewer artifacts than the SPAIR technique. In terms of the evaluation of the characteristics of TAO lesions, the former showed a stronger ability to evaluate TAO inflammation and higher sensitivity and specificity for the active staging of TAO than the latter. Dixon technology provides an excellent fat suppression technique for MRI examination of TAO patients, and also provides a certain research basis for the next step to explore the imaging criteria for TAO activity assessment and efficacy prediction.

## Data availability statement

The raw data supporting the conclusions of this article will be made available by the authors, without undue reservation.

## Ethics statement

The studies involving human participants were reviewed and approved by the Ethics Committee of Shunde Hospital of Southern Medical University (KYLS20220123). Written informed consent to participate in this study was provided by the participants’ legal guardian/next of kin.

## Author contributions

KH, XL, YL, QH, BG, FO, YO, CS, and HC contributed to the study’s conception and design. Clinical data collection and analysis were performed by KH, YL, and CS. Image scanning and analysis were performed by XL and YO. Conception, design, and administrative support were performed by QH, BG, and FO. The first draft of the manuscript was written by KH and HC. All authors contributed to the article and approved the submitted version.

## Funding

This research was supported by the Medical Scientific and Technology Research Foundation of Guangdong Province of China (B2022185), Foshan self-raised funds of science and technology plan projects (2220001003987) and the Research Initiation Program of Shunde Hospital, Southern Medical University (the First People’s Hospital of Shunde District, Foshan) (SRSP2021037).

## Conflict of interest

The authors declare that the research was conducted in the absence of any commercial or financial relationships that could be construed as a potential conflict of interest.

## Publisher’s note

All claims expressed in this article are solely those of the authors and do not necessarily represent those of their affiliated organizations, or those of the publisher, the editors and the reviewers. Any product that may be evaluated in this article, or claim that may be made by its manufacturer, is not guaranteed or endorsed by the publisher.
